# Epidemiology of breast cancer in Cyprus: Data on newly diagnosed cases and survival rates

**DOI:** 10.1016/j.dib.2018.05.042

**Published:** 2018-05-19

**Authors:** Pampina Pilavaki, George Giallouros, Anneza I. Yiallourou, Katerina Pantavou, Yiola Marcou, Anna Demetriou, Vasos Scoutellas, Georgios K. Nikolopoulos

**Affiliations:** a Medical School, University of Cyprus, Nicosia, Cyprus; b Bank of Cyprus Oncology Center, Nicosia, Cyprus; c Health Monitoring Unit, Ministry of Health, Cyprus

## Abstract

This article presents analyzed data on new diagnoses and mortality of breast cancer, between 2005 and 2013, in the Republic of Cyprus. New diagnoses are presented by demographic and clinical/histological variables that include cancer grade, behaviour, stage, and histological type at diagnosis (always as a primary site). Breast cancer-related deaths are presented by gender. Net survival rates based on cohort and period methods are presented by age group, cancer grade, behaviour, and stage at diagnosis, for all cases and for cases of Greek-Cypriot ethnicity. The unprocessed data of the Cyprus Cancer Registry were provided by the Health Monitoring Unit of the Ministry of Health of the Republic of Cyprus.

**Specifications Table**

**Table:** 

Subject area	*Medicine*
More specific subject area	*Epidemiology*
Type of data	*Tables*
How data was acquired	*Unprocessed primary data*
Data format	*Analyzed*
Experimental factors	*Duplicates were deleted from the dataset*
Experimental features	*The unprocessed data of the Cancer Registry were provided by the Health Monitoring Unit of the Ministry of Health of the Republic of Cyprus in an electronic form and were analyzed using STATA version 14 (StataCorp LP, College Station, Texas, USA)*
Data source location	*Republic of Cyprus*
Data accessibility	*Data are provided within this article*

**Value of the data**

• The presented data can be used as reference for the Republic of Cyprus for the years 2005–2013 and can be compared with those of other studies that might be published in the future.
• The data can be included in analyses to better understand the epidemiology of breast cancer.
• The data can be used for developing policies related to breast cancer prevention.

## 1 Data

This article includes analyzed data on breast cancer cases obtained from the Cyprus Cancer Registry in the Health Monitoring Unit of the Ministry of Health of the Republic of Cyprus for the period 2005–2013. Demographic and clinical/histological parameters of newly diagnosed breast cancer cases are shown in Table 1, Table 2, Table 3, Table 4, Table 5. Table 6 presents the classification of histological types and their subtypes (always as primary sites) used for the analysis of data. Breast cancer mortality per year can be found in Table 7. Net survival rates for breast cancer (Table 8, Table 9, Table 10, Table 11, Table 12, Table 13, Table 14, Table 15) are presented for 2004–2008 and 2009–2013.

**Table 1:** Demographic characteristics of newly diagnosed breast cancer cases (*N* = 4769) by year of diagnosis (2005–2013).

		**2005**		**2006**		**2007**		**2008**		**2009**		**2010**		**2011**		**2012**		**2013**		**Total**	
**Gender** [*N* (%)]	Female	452	(98.9)	433	(98.2)	535	(98.9)	510	(98.5)	476	(99.2)	549	(99.5)	602	(99.2)	562	(99.1)	603	(99.5)	4722	(99.0)
	Male	5	(1.1)	8	(1.8)	6	(1.1)	8	(1.5)	4	(0.8)	3	(0.5)	5	(0.8)	5	(0.9)	3	(0.5)	47	(1.0)
**Age**	Median (IQR)	57	(46–67)	58	(47–68)	58	(48–68)	58	(49–69)	58	(49–68)	58	(49–69)	59	(49–68)	61	(49–69)	60	(50–69)	59	(49–59)
																					
**Ethnicity** [*N* (%)]	Greek-Cypriot	398	(87.1)	372	(84.4)	460	(85.0)	435	(84.0)	411	(85.6)	467	(84.6)	508	(83.7)	475	(83.8)	524	(86.5)	4050	(85.0)
	Non-Greek-Cypriot^a^	59	(12.9)	65	(14.7)	76	(14.1)	77	(14.9)	65	(13.6)	81	(14.7)	83	(13.7)	73	(12.9)	71	(11.7)	650	(13.6)
	Unknown	0	(0.0)	4	(0.9)	5	(0.9)	6	(1.1)	4	(0.8)	4	(0.7)	16	(2.6)	19	(3.3)	11	(1.8)	69	(1.4)
																					
**Marital Status** [*N* (%)]	Single	31	(6.8)	23	(5.2)	28	(5.2)	26	(5.0)	26	(5.4)	30	(5.4)	38	(6.3)	34	(6.0)	31	(5.1)	267	(5.6)
	Married	307	(67.2)	298	(67.6)	383	(70.8)	369	(71.3)	348	(72.5)	383	(69.4)	410	(67.5)	361	(63.7)	399	(65.9)	3258	(68.3)
	Divorced/Separated	22	(4.8)	32	(7.2)	34	(6.3)	25	(4.8)	21	(4.4)	27	(4.9)	32	(5.3)	43	(7.6)	36	(5.9)	272	(5.7)
	Widowed	57	(12.5)	59	(13.4)	61	(11.3)	57	(11.0)	46	(9.6)	62	(11.2)	66	(10.9)	64	(11.3)	60	(9.9)	532	(11.2)
	Unknown	40	(8.7)	29	(6.6)	35	(6.4)	41	(7.9)	39	(8.1)	50	(9.1)	61	(10.0)	65	(11.4)	80	(13.2)	440	(9.2)
																					
**Smoking History** [*N* (%)]	Yes	45	(9.8)	54	(12.2)	82	(15.2)	60	(11.6)	34	(7.1)	65	(11.8)	80	(13.2)	83	(14.6)	59	(9.7)	562	(11.8)
	No	164	(35.9)	137	(31.1)	188	(34.7)	213	(41.1)	214	(44.6)	243	(44.0)	259	(42.7)	247	(43.6)	241	(39.8)	1906	(40.0)
	Unknown	248	(54.3)	250	(56.7)	271	(50.1)	245	(47.3)	232	(48.3)	244	(44.2)	268	(44.1)	237	(41.8)	306	(50.5)	2301	(48.2)
																					
**Birthplace**^b^ [*N* (%)]	Ammochostos	71	(15.5)	57	(12.9)	92	(17.0)	61	(11.8)	92	(19.2)	66	(12.0)	94	(15.5)	76	(13.4)	92	(15.2)	701	(14.7)
	Kyrenia	18	(3.9)	16	(3.6)	34	(6.3)	12	(2.3)	13	(2.7)	20	(3.6)	20	(3.3)	18	(3.2)	19	(3.1)	170	(3.6)
	Larnaca	43	(9.4)	32	(7.3)	43	(8.0)	30	(5.8)	37	(7.7)	35	(6.3)	42	(6.9)	45	(7.9)	50	(8.3)	357	(7.5)
	Nicosia	118	(25.8)	130	(29.5)	143	(26.4)	157	(30.3)	147	(30.6)	174	(31.5)	173	(28.5)	177	(31.2)	200	(33.0)	1419	(29.8)
	Limassol	66	(14.4)	56	(12.7)	79	(14.6)	81	(15.6)	59	(12.3)	68	(12.3)	83	(13.7)	72	(12.7)	72	(11.9)	636	(13.3)
	Pafos	35	(7.7)	33	(7.5)	30	(5.6)	36	(7.0)	27	(5.6)	47	(8.5)	42	(6.9)	27	(4.8)	31	(5.1)	308	(6.5)
	Unknown	106	(23.3)	117	(26.5)	120	(22.1)	141	(27.2)	105	(21.9)	142	(25.8)	153	(25.2)	152	(26.8)	142	(23.4)	1178	(24.6)

a Non-Greek Cypriots include Armenians, Maronites, European Union (EU) foreigners, and non-EU foreigners.

b Birthplace refers to one of the six districts of the Republic of Cyprus.

**Table 2:** Cancer grade of newly diagnosed breast cancer cases (*N* = 4700) by ethnicity and year of diagnosis (2005–2013).

	**Cancer grade**	**2005**		**2006**		**2007**		**2008**		**2009**		**2010**		**2011**		**2012**		**2013**		**Total**	
**Greek-Cypriot** [*N* (%)]	I; Well Differentiated	32	(8.0)	36	(9.7)	59	(12.8)	33	(7.6)	35	(8.5)	36	(7.7)	28	(5.5)	38	(8.0)	30	(5.7)	327	(8.1)
	II; Moderately Differentiated	181	(45.5)	161	(43.3)	200	(43.5)	173	(39.8)	182	(44.3)	205	(43.9)	236	(46.5)	209	(44.0)	264	(50.4)	1811	(44.7)
	III; Poorly Differentiated	97	(24.4)	98	(26.3)	110	(23.9)	159	(36.5)	135	(32.8)	144	(30.8)	160	(31.5)	163	(34.3)	143	(27.3)	1209	(29.8)
	Undifferentiated/Unknown	88	(22.1)	77	(20.7)	91	(19.8)	70	(16.1)	59	(14.4)	82	(17.6)	84	(16.5)	65	(13.7)	87	(16.6)	703	(17.4)
																					
**Non-Greek- Cypriot**^a^ [*N* (%)]	I; Well Differentiated	3	(5.1)	3	(4.6)	2	(2.6)	5	(6.4)	2	(3.1)	4	(4.9)	1	(1.2)	4	(5.5)	3	(4.2)	27	(4.2)
	II; Moderately Differentiated	30	(50.9)	25	(38.5)	29	(38.2)	32	(41.6)	28	(43.1)	38	(46.9)	41	(49.4)	30	(41.1)	29	(40.8)	282	(43.4)
	III; Poorly Differentiated	11	(18.6)	23	(35.4)	26	(34.2)	32	(41.6)	30	(46.1)	27	(33.4)	33	(39.8)	30	(41.1)	33	(46.5)	245	(37.7)
	Undifferentiated/Unknown	15	(25.4)	14	(21.5)	19	(25.0)	8	(10.4)	5	(7.7)	12	(14.8)	8	(9.6)	9	(12.3)	6	(8.5)	96	(14.7)
																					
**Total** [*N* (%)]	I; Well Differentiated	35	(7.7)	39	(8.9)	61	(11.4)	38	(7.4)	37	(7.8)	40	(7.3)	29	(4.9)	42	(7.7)	33	(5.6)	354	(7.5)
	II; Moderately Differentiated	211	(46.2)	186	(42.6)	229	(42.7)	205	(40.0)	210	(44.1)	243	(44.2)	277	(46.9)	239	(43.5)	293	(49.2)	2093	(44.5)
	III; Poorly Differentiated	108	(23.6)	121	(27.7)	136	(25.4)	191	(37.4)	165	(34.7)	171	(31.3)	193	(32.6)	193	(35.3)	176	(29.6)	1454	(30.9)
	Undifferentiated/Unknown	103	(22.5)	91	(20.8)	110	(20.5)	78	(15.2)	64	(13.4)	94	(17.2)	92	(15.6)	74	(13.5)	93	(15.6)	799	(17.0)

a Non-Greek Cypriots include Armenians, Maronites, European Union (EU) foreigners, and non-EU foreigners.

**Table 3:** Cancer behaviour of newly diagnosed breast cancer cases (*N* = 4700) by ethnicity and year of diagnosis (2005–2013).

	**Cancer behaviour**	**2005**		**2006**		**2007**		**2008**		**2009**		**2010**		**2011**		**2012**		**2013**		**Total**	
**Greek-Cypriot** [*N* (%)]	In situ	33	(8.3)	19	(5.1)	48	(10.4)	42	(9.7)	39	(9.5)	43	(9.2)	47	(9.2)	38	(8.0)	57	(10.9)	366	(9.0)
	Invasive	365	(91.7)	353	(94.9)	412	(89.6)	393	(90.3)	372	(90.5)	424	(90.8)	461	(90.8)	437	(92.0)	468	(89.1)	3685	(91.0)
																					
**Non-Greek-Cypriot**^a^ [*N* (%)]	In situ	5	(8.5)	2	(3.1)	5	(6.6)	2	(2.6)	1	(1.5)	6	(7.4)	2	(2.4)	4	(5.5)	2	(2.8)	29	(4.5)
	Invasive	54	(91.5)	63	(96.9)	71	(93.4)	75	(97.4)	64	(98.5)	75	(92.6)	81	(97.6)	69	(94.5)	69	(97.2)	621	(95.5)
																					
**Total** [N (%)]	In situ	38	(8.3)	21	(4.8)	53	(9.9)	44	(8.6)	40	(8.4)	49	(8.9)	49	(8.3)	42	(7.7)	59	(9.7)	395	(8.4)
	Invasive	419	(91.7)	416	(95.2)	483	(90.1)	468	(91.4)	436	(91.6)	499	(91.1)	542	(91.7)	506	(92.3)	537	(90.3)	4306	(91.6)

a Non-Greek Cypriots include Armenians, Maronites, European Union (EU) foreigners, and non-EU foreigners.

**Table 4:** Cancer stage of newly diagnosed breast cancer cases (*N* = 4700) by ethnicity and year of diagnosis (2005–2013).

	**Cancer stage**	**2005**		**2006**		**2007**		**2008**		**2009**		**2010**		**2011**		**2012**		**2013**		**Total**	
**Greek-Cypriot** [*N* (%)]	Distant metastatic	16	(4.0)	17	(4.6)	25	(5.4)	23	(5.3)	18	(4.4)	17	(3.6)	19	(3.7)	15	(3.2)	9	(1.7)	159	(3.9)
	In situ	33	(8.3)	19	(5.1)	48	(10.5)	42	(9.7)	39	(9.5)	43	(9.2)	47	(9.3)	38	(8.0)	57	(10.9)	366	(9.0)
	Locoregional invasive	320	(80.4)	302	(81.2)	340	(73.9)	328	(75.4)	315	(76.6)	353	(75.6)	383	(75.4)	365	(76.8)	396	(75.6)	3102	(76.6)
	Unknown	29	(7.3)	34	(9.1)	47	(10.2)	42	(9.6)	39	(9.5)	54	(11.6)	59	(11.6)	57	(12.0)	62	(11.8)	423	(10.4)
																					
**Non-Greek-Cypriot**^a^ [*N* (%)]	Distant metastatic	1	(1.7)	3	(4.6)	4	(5.3)	5	(6.5)	3	(4.6)	6	(7.4)	2	(2.4)	6	(8.2)	2	(2.8)	32	(5.0)
	In situ	5	(8.5)	2	(3.1)	5	(6.6)	2	(2.6)	1	(1.5)	6	(7.4)	2	(2.4)	4	(5.5)	2	(2.8)	29	(4.5)
	Locoregional invasive	49	(83.0)	55	(84.6)	57	(75.0)	60	(77.9)	51	(78.5)	64	(79.0)	67	(80.7)	54	(74.0)	55	(77.5)	512	(78.8)
	Unknown	4	(6.8)	5	(7.7)	10	(13.1)	10	(13.0)	10	(15.4)	5	(6.2)	12	(14.5)	9	(12.3)	12	(16.9)	77	(11.7)
																					
**Total** [*N* (%)]	Distant metastatic	17	(3.7)	20	(4.6)	29	(5.4)	28	(5.5)	21	(4.4)	23	(4.2)	21	(3.6)	21	(3.8)	11	(1.9)	191	(4.1)
	In situ	38	(8.3)	21	(4.8)	53	(9.9)	44	(8.6)	40	(8.4)	49	(8.9)	49	(8.3)	42	(7.7)	59	(9.9)	395	(8.4)
	Locoregional invasive	369	(80.8)	357	(81.7)	397	(74.1)	388	(75.7)	366	(76.9)	417	(76.1)	450	(76.1)	419	(76.5)	451	(75.8)	3614	(76.9)
	Unknown	33	(7.2)	39	(8.9)	57	(10.6)	52	(10.2)	49	(10.3)	59	(10.8)	71	(12.0)	66	(12.0)	74	(12.4)	500	(10.6)

a Non-Greek Cypriots include Armenians, Maronites, European Union (EU) foreigners, and non-EU foreigners.

**Table 5:** Cancer histologic morphological type of newly diagnosed breast cancer cases (*N* = 4700) by ethnicity and year of diagnosis (2005–2013).

	**Histologic morphological type**	**2005**		**2006**		**2007**		**2008**		**2009**		**2010**		**2011**		**2012**		**2013**		**Total**	
**Greek-Cypriot** [*N* (%)]	Adenoid cystic carcinoma	0	(0.0)	0	(0.0)	1	(0.2)	0	(0.0)	0	(0.0)	0	(0.0)	0	(0.0)	0	(0.0)	0	(0.0)	1	(0.0)
	Carcinoma with apocrine features	0	(0.0)	0	(0.0)	0	(0.0)	0	(0.0)	0	(0.0)	2	(0.4)	0	(0.0)	0	(0.0)	1	(0.2)	3	(0.1)
	Carcinoma with medullary features	2	(0.5)	1	(0.3)	3	(0.7)	0	(0.0)	0	(0.0)	1	(0.2)	0	(0.0)	0	(0.0)	2	(0.4)	9	(0.2)
	Cribriform carcinoma	0	(0.0)	0	(0.0)	2	(0.4)	0	(0.0)	0	(0.0)	1	(0.2)	0	(0.0)	0	(0.0)	2	(0.4)	5	(0.1)
	Inflammatory carcinoma	0	(0.0)	0	(0.0)	1	(0.2)	0	(0.0)	0	(0.0)	0	(0.0)	0	(0.0)	0	(0.0)	0	(0.0)	1	(0.0)
	Invasive carcinoma of no special type	291	(73.1)	256	(68.8)	338	(73.5)	354	(81.4)	328	(79.8)	353	(75.6)	406	(79.9)	381	(80.2)	416	(79.4)	3123	(77.1)
	Invasive lobular carcinoma	42	(10.5)	50	(13.4)	41	(8.9)	38	(8.7)	51	(12.4)	56	(12.0)	49	(9.7)	58	(12.2)	60	(11.5)	445	(11.0)
	Mesenchymal tumours	0	(0.0)	0	(0.0)	1	(0.2)	1	(0.2)	0	(0.0)	0	(0.0)	0	(0.0)	1	(0.2)	0	(0.0)	3	(0.1)
	Metaplastic carcinoma	0	(0.0)	0	(0.0)	2	(0.4)	0	(0.0)	0	(0.0)	0	(0.0)	4	(0.8)	1	(0.2)	0	(0.0)	7	(0.2)
	Mucinous carcinoma	7	(1.8)	6	(1.6)	12	(2.6)	8	(1.8)	1	(0.2)	9	(1.9)	4	(0.8)	8	(1.7)	12	(2.3)	67	(1.7)
	Paget׳s disease	2	(0.5)	3	(0.8)	2	(0.4)	1	(0.2)	2	(0.5)	2	(0.4)	3	(0.6)	2	(0.4)	2	(0.4)	19	(0.5)
	Phyllodes tumour	3	(0.7)	2	(0.5)	2	(0.4)	0	(0.0)	1	(0.2)	0	(0.0)	0	(0.0)	1	(0.2)	1	(0.2)	10	(0.3)
	Rare variants	42	(10.6)	48	(12.8)	47	(10.3)	28	(6.4)	22	(5.4)	37	(7.9)	37	(7.3)	21	(4.4)	24	(4.5)	306	(7.5)
	Tubular carcinoma	9	(2.3)	6	(1.6)	8	(1.8)	5	(1.3)	6	(1.5)	6	(1.4)	5	(0.9)	2	(0.5)	4	(0.7)	51	(1.2)
																					
**Non-Greek-Cypriot**^a^ [*N* (%)]	Adenoid cystic carcinoma	0	(0.0)	0	(0.0)	0	(0.0)	0	(0.0)	0	(0.0)	0	(0.0)	0	(0.0)	0	(0.0)	0	(0.0)	0	(0.0)
	Carcinoma with apocrine features	0	(0.0)	0	(0.0)	0	(0.0)	0	(0.0)	1	(1.5)	0	(0.0)	0	(0.0)	0	(0.0)	0	(0.0)	1	(0.2)
	Carcinoma with medullary features	0	(0.0)	0	(0.0)	1	(1.3)	0	(0.0)	1	(1.5)	0	(0.0)	0	(0.0)	0	(0.0)	0	(0.0)	2	(0.3)
	Cribriform carcinoma	0	(0.0)	0	(0.0)	0	(0.0)	0	(0.0)	0	(0.0)	0	(0.0)	0	(0.0)	0	(0.0)	0	(0.0)	0	(0.0)
	Inflammatory carcinoma	0	(0.0)	0	(0.0)	1	(1.3)	0	(0.0)	0	(0.0)	0	(0.0)	0	(0.0)	0	(0.0)	0	(0.0)	1	(0.2)
	Invasive carcinoma of no special type	50	(84.8)	57	(87.7)	59	(77.6)	60	(77.9)	51	(78.5)	69	(85.2)	72	(86.8)	63	(86.3)	56	(78.9)	537	(82.5)
	Invasive lobular carcinoma	6	(10.1)	4	(6.1)	5	(6.7)	10	(13.0)	7	(10.8)	6	(7.5)	4	(4.8)	5	(6.8)	8	(11.3)	55	(8.4)
	Mesenchymal tumours	0	(0.0)	0	(0.0)	0	(0.0)	0	(0.0)	0	(0.0)	0	(0.0)	0	(0.0)	0	(0.0)	0	(0.0)	0	(0.0)
	Metaplastic carcinoma	0	(0.0)	0	(0.0)	0	(0.0)	1	(1.3)	0	(0.0)	0	(0.0)	0	(0.0)	0	(0.0)	0	(0.0)	1	(0.2)
	Mucinous carcinoma	1	(1.7)	0	(0.0)	1	(1.3)	0	(0.0)	2	(3.1)	1	(1.2)	0	(0.0)	1	(1.4)	3	(4.2)	9	(1.4)
	Paget׳s disease	0	(0.0)	0	(0.0)	0	(0.0)	0	(0.0)	0	(0.0)	1	(1.2)	1	(1.2)	1	(1.4)	0	(0.0)	3	(0.5)
	Phyllodes tumour	0	(0.0)	0	(0.0)	1	(1.3)	0	(0.0)	0	(0.0)	0	(0.0)	0	(0.0)	0	(0.0)	0	(0.0)	1	(0.2)
	Rare variants	2	(3.4)	4	(6.2)	8	(10.5)	5	(6.5)	3	(4.6)	4	(4.9)	6	(7.2)	3	(4.1)	3	(4.2)	38	(5.8)
	Tubular carcinoma	0	(0.0)	0	(0.0)	0	(0.0)	1	(1.3)	0	(0.0)	0	(0.0)	0	(0.0)	0	(0.0)	1	(1.4)	2	(0.3)
																					
**Total** [*N* (%)]	Adenoid cystic carcinoma	0	(0.0)	0	(0.0)	1	(0.2)	0	(0.0)	0	(0.0)	0	(0.0)	0	(0.0)	0	(0.0)	0	(0.0)	1	(0.0)
	Carcinoma with apocrine features	0	(0.0)	0	(0.0)	0	(0.0)	0	(0.0)	1	(0.2)	2	(0.4)	0	(0.0)	0	(0.0)	1	(0.2)	4	(0.1)
	Carcinoma with medullary features	2	(0.4)	1	(0.2)	4	(0.7)	0	(0.0)	1	(0.2)	1	(0.2)	0	(0.0)	0	(0.0)	2	(0.3)	11	(0.2)
	Cribriform carcinoma	0	(0.0)	0	(0.0)	2	(0.4)	0	(0.0)	0	(0.0)	1	(0.2)	0	(0.0)	0	(0.0)	2	(0.3)	5	(0.1)
	Inflammatory carcinoma	0	(0.0)	0	(0.0)	2	(0.4)	0	(0.0)	0	(0.0)	0	(0.0)	0	(0.0)	0	(0.0)	0	(0.0)	2	(0.0)
	Invasive carcinoma of no special type	341	(74.6)	313	(71.6)	397	(74.1)	414	(80.9)	379	(79.6)	422	(77.0)	478	(80.9)	444	(81.0)	482	(79.5)	3660	(77.9)
	Invasive lobular carcinoma	48	(10.5)	54	(12.3)	46	(8.5)	48	(9.3)	58	(12.2)	62	(11.2)	53	(8.9)	63	(11.4)	68	(11.3)	500	(10.7)
	Mesenchymal tumours	0	(0.0)	0	(0.0)	1	(0.2)	1	(0.2)	0	(0.0)	0	(0.0)	0	(0.0)	1	(0.2)	0	(0.0)	3	(0.1)
	Metaplastic carcinoma	0	(0.0)	0	(0.0)	2	(0.4)	1	(0.2)	0	(0.0)	0	(0.0)	4	(0.7)	1	(0.2)	0	(0.0)	8	(0.2)
	Mucinous carcinoma	8	(1.8)	6	(1.4)	13	(2.4)	8	(1.5)	3	(0.6)	10	(1.8)	4	(0.7)	9	(1.6)	15	(2.5)	76	(1.6)
	Paget׳s disease	2	(0.4)	3	(0.7)	2	(0.4)	1	(0.2)	2	(0.4)	3	(0.6)	4	(0.7)	3	(0.6)	2	(0.3)	22	(0.5)
	Phyllodes tumour	3	(0.7)	2	(0.5)	3	(0.6)	0	(0.0)	1	(0.2)	0	(0.0)	0	(0.0)	1	(0.2)	1	(0.2)	11	(0.2)
	Rare variants	44	(9.6)	52	(11.9)	55	(10.2)	33	(6.5)	25	(5.3)	41	(7.5)	43	(7.2)	24	(4.4)	28	(4.6)	344	(7.3)
	Tubular carcinoma	9	(2.0)	6	(1.4)	8	(1.5)	6	(1.2)	6	(1.3)	6	(1.1)	5	(0.9)	2	(0.4)	5	(0.8)	53	(1.1)

a Non-Greek Cypriots include Armenians, Maronites, European Union (EU) foreigners, and non-EU foreigners.

**Table 6:** Histological classification of breast cancer types and subtypes (based on International Classification of Diseases for Oncology morphological codes, ICD-O-3 [2]).

**Histologic type**		**ICD-O-3 morphology code**
**Categories**	**Subtypes**	
Adenoid cystic carcinoma		8200/3
Carcinoma with apocrine features		8401/3
Carcinoma with medullary features	Atypical medullary carcinoma	8513/3
	Medullary carcinoma, NOS^a^	8510/3
Cribriform carcinoma		8201/3
Inflammatory carcinoma		8530/3
Invasive carcinoma of no special type	Duct carcinoma	8500/3
	Infiltrating duct mixed with other types of carcinoma	8523/3
	Infiltrating ductular carcinoma	8521/3
	Solid carcinoma, NOS^a^	8230/3
Invasive lobular carcinoma	Infiltrating lobular mixed with other types of carcinoma	8524/3
	Lobular carcinoma, NOS^a^	8520/3
Mesenchymal tumours	Fibrosarcoma, NOS^a^	8810/3
	Haemangiosarcoma	9120/3
	Liposarcoma, NOS^a^	8850/3
Metaplastic carcinoma	Metaplastic carcinoma, NOS^a^	8575/3
	Signet ring cell carcinoma	8490/3
	Spindle cell carcinoma, NOS^a^	8032/3
	Squamous cell carcinoma, NOS^a^	8070/3
Mucinous carcinoma	Mucin-producing adenocarcinoma	8481/3
	Mucinous adenocarcinoma	8480/3
Paget׳s disease	Paget disease and infiltrating duct carcinoma of breast	8541/3
	Paget disease and intraductal carcinoma of breast	8543/3
	Paget disease, mammary	8540/3
Phyllodes tumour	Phyllodes tumour, malignant	9020/3
Rare variants	Granular cell tumour, malignant	9580/3
	Neuroendocrine carcinoma, NOS^a^	8246/3
	Papillary adenocarcinoma, NOS^a^	8260/3
	Papillary carcinoma, NOS^a^	8050/3
	Mixed cell adenocarcinoma	8323/3
	Carcinoma, undifferentiated, NOS^a^	8020/3
Tubular carcinoma	Tubular adenocarcinoma	8211/3

a NOS stands for Not Otherwise Specified.

**Table 7:** Breast cancer-related deaths (*N* = 930) by gender and year of diagnosis for the period 2005–2013. The numbers of deaths attributable to breast cancer per 1000 deaths are shown in parentheses.

	**Gender**	**2005**		**2006**		**2007**		**2008**		**2009**		**2010**		**2011**		**2012**		**2013**		**Overall**	
**Breast Cancer Deaths** [*N* (N per 1000 deaths)]	Female	72	(27.8)	81	(32.4)	92	(35.8)	94	(38.3)	106	(44.1)	110	(46.4)	118	(45.0)	111	(41.4)	131	(54.9)	915	(40.5)
	Male	0	(0.0)	2	(0.8)	1	(0.4)	3	(1.1)	3	(1.1)	1	(0.4)	2	(0.7)	3	(1.0)	0	(0.0)	15	(0.6)
	Total	72	(13.3)	83	(16.2)	93	(17.3)	97	(18.7)	109	(21.0)	111	(21.8)	120	(22.3)	114	(20.1)	131	(24.8)	930	(19.5)

**Table 8:** Five-year net survival rates of all breast cancer cases by age group.

**Time period**	**Age group**															
**Survival analysis method**	**19–29**		**30–39**		**40–49**		**50–59**		**60–69**		**70–79**		**80-**		**Overall**	
**Net Survival for 2004–2008 (95% CI**^a^**) - Cohort method**^b^	0.85	(0.66–1.04)	0.88	(0.81–0.94)	0.91	(0.88–0.94)	0.87	(0.84–0.90)	0.84	(0.81–0.88)	0.69	(0.64–0.75)	0.36	(0.27–0.45)	0.82	(0.80–0.83)
**Net Survival for 2009–2013 (95% CI**^a^**) - Period method**^c^	0.80	(0.59–1.00)	0.93	(0.90–0.97)	0.94	(0.93–0.96)	0.90	(0.89–0.92)	0.89	(0.86–0.91)	0.79	(0.75–0.82)	0.60	(0.54–0.66)	0.87	(0.86–0.88)

a CI: Confidence Interval.

b The Cohort method refers to people diagnosed in 2004–2008 (*N* = 2296) who were followed up at least five years after their diagnosis.

c The Period method refers to survival experiences in 2009–2013 of people diagnosed between 2004–2013 (*N* = 4324).

**Table 9:** Five-year net survival rates of all breast cancer cases by different cancer grade at diagnosis.

**Time period**	**Cancer Grade**									
**Survival analysis method**	**I; Well Differentiated**		**II; Moderately Differentiated**		**III; Poorly Differentiated**		**Undifferentiated/Unknown**		**Overall**	
**Net Survival for 2004–2008 (95% CI**^a^**) - Cohort method**^b^	0.91	(0.86–0.95)	0.86	(0.83–0.88)	0.73	(0.69–0.77)	0.79	(0.74–0.84)	0.82	(0.80–0.83)
**Net Survival for 2009–2013 (95% CI**^a^**) - Period method**^c^	0.92	(0.88–0.95)	0.89	(0.87–0.90)	0.84	(0.82–0.86)	0.88	(0.84–0.91)	0.87	(0.86–0.88)

a CI: Confidence Interval.

b The Cohort method refers to people diagnosed in 2004–2008 (*N* = 2296) who were followed up at least five years after their diagnosis.

c The Period method refers to survival experiences in 2009–2013 of people diagnosed between 2004–2013 (*N* = 4324).

**Table 10:** Five-year net survival rates of all breast cancer cases by cancer behaviour at diagnosis.

**Time period**	**Cancer Behaviour**					
**Survival analysis method**	**Invasive**		**Ιn situ**		**Overall**	
**Net Survival for 2004–2008 (95% CI**^a^**) - Cohort method**^b^	0.81	(0.79–0.83)	0.99	(0.96–1.01)	0.82	(0.80–0.83)
**Net Survival for 2009–2013 (95% CI**^a^**) - Period method**^c^	0.86	(0.85–0.88)	0.98	(0.96–1.00)	0.87	(0.86–0.88)

a CI: Confidence Interval.

b The Cohort method refers to people diagnosed in 2004–2008 (*N* = 2296) who were followed up at least five years after their diagnosis.

c The Period method refers to survival experiences in 2009–2013 of people diagnosed between 2004–2013 (*N* = 4324).

**Table 11:** Five-year net survival rates of all breast cancer cases by cancer stage at diagnosis.

**Time period**	**Cancer stage**									
**Survival analysis method**	**Distant metastatic**		**Ιn situ**		**Locoregional invasive**		**Unknown**		**Overall**	
**Net Survival for 2004–2008 (95% CI**^a^**) - Cohort method**^b^	0.34	(0.24–0.43)	0.99	(0.96–1.01)	0.85	(0.84–0.87)	0.56	(0.46–0.66)	0.82	(0.80–0.83)
**Net Survival for 2009–2013 (95% CI**^a^**) - Period method**^c^	0.49	(0.41–0.58)	0.99	(0.98–1.00)	0.89	(0.88–0.90)	0.73	(0.68–0.79)	0.87	(0.86–0.88)

a CI: Confidence Interval.

b The Cohort method refers to people diagnosed in 2004–2008 (*N* = 2296) who were followed up at least five years after their diagnosis.

c The Period method refers to survival experiences in 2009–2013 of people diagnosed between 2004–2013 (*N* = 4324).

**Table 12:** Five-year net survival rates of Greek-Cypriot breast cancer cases by age group.

**Time period**	**Age group**															
**Survival analysis method**	**19–29**		**30–39**		**40–49**		**50–59**		**60–69**		**70–79**		**80–**		**Overall**	
**Net Survival for 2004–2008 (95% CI**^a^**) - Cohort method**^b^	0.83	(0.61–1.04)	0.86	(0.79–0.93)	0.90	(0.87–0.93)	0.88	(0.84–0.91)	0.85	(0.81–0.89)	0.69	(0.63–0.75)	0.37	(0.27–0.46)	0.81	(0.79–0.83)
**Net Survival for 2009–2013 (95% CI**^a^**) - Period method**^c^	0.86	(0.68–1.03)	0.93	(0.89–0.97)	0.94	(0.92–0.96)	0.91	(0.89–0.93)	0.89	(0.87–0.91)	0.78	(0.74–0.81)	0.59	(0.53–0.66)	0.87	(0.86–0.88)

a CI: Confidence Interval.

b The Cohort method refers to people diagnosed in 2004–2008 (*N* = 1982) who were followed up at least five years after their diagnosis.

c The Period method refers to survival experiences in 2009–2013 of people diagnosed between 2004–2013 (*N* = 3735).

**Table 13:** Five-year net survival rates of Greek-Cypriot breast cancer cases by cancer grade at diagnosis.

**Time period**	**Cancer grade**									
**Survival analysis method**	**I; Well Differentiated**		**II; Moderately Differentiated**		**III; Poorly Differentiated**		**Undifferentiated/Unknown**		**Overall**	
**Net Survival for 2004–2008 (95% CI**^a^**) - Cohort method**^b^	0.91	(0.86–0.95)	0.85	(0.83–0.88)	0.73	(0.69–0.77)	0.79	(0.74–0.85)	0.81	(0.79–0.83)
**Net Survival for 2009–2013 (95% CI**^a^**) - Period method**^c^	0.92	(0.88–0.95)	0.88	(0.87–0.90)	0.83	(0.81–0.86)	0.88	(0.84–0.91)	0.87	(0.86–0.88)

a CI: Confidence Interval.

b The Cohort method refers to people diagnosed in 2004–2008 (*N* = 1982) who were followed up at least five years after their diagnosis.

c The Period method refers to survival experiences in 2009–2013 of people diagnosed between 2004–2013 (*N* = 3735).

**Table 14:** Five-year net survival rates of Greek-Cypriot breast cancer cases by cancer behaviour at diagnosis.

**Time period**	**Cancer behaviour**					
**Survival analysis method**	**Invasive**		**In situ**		**Overall**	
**Net Survival for 2004–2008 (95% CI**^a^**) - Cohort method**^b^	0.80	(0.78–0.82)	0.98	(0.95–1.01)	0.81	(0.79–0.83)
**Net Survival for 2009–2013 (95% CI**^a^**) - Period method**^c^	0.86	(0.85–0.87)	0.98	(0.96–1.00)	0.87	(0.86–0.88)

a CI: Confidence Interval.

b The Cohort method refers to people diagnosed in 2004–2008 (*N* = 1982) who were followed up at least five years after their diagnosis.

c The Period method refers to survival experiences in 2009–2013 of people diagnosed between 2004–2013 (*N* = 3735).

**Table 15:** Five-year net survival rates of Greek-Cypriot breast cancer cases by cancer stage at diagnosis.

**Time period**	**Cancer stage**									
**Survival analysis method**	**Distant metastatic**		**In situ**		**Locoregional invasive**		**Unknown**		**Overall**	
**Net Survival for 2004–2008 (95% CI**^a^**) - Cohort method**^b^	0.33	(0.24–0.43)	0.98	(0.95–1.01)	0.85	(0.83–0.87)	0.50	(0.39–0.61)	0.81	(0.79–0.83)
**Net Survival for 2009–2013 (95% CI**^a^**) - Period method**^c^	0.47	(0.39–0.56)	0.98	(0.96–1.00)	0.89	(0.88–0.90)	0.72	(0.66–0.78)	0.87	(0.86–0.88)

a CI: Confidence Interval.

b The Cohort method refers to people diagnosed in 2004–2008 (*N* = 1982) who were followed up at least five years after their diagnosis.

c The Period method refers to survival experiences in 2009–2013 of people diagnosed between 2004–2013 (*N* = 3735).

Table 1 shows counts and percentages of newly diagnosed breast cancer cases by gender, age, ethnicity (Greek-Cypriot/Non-Greek-Cypriot/Unknown), marital status at the time of diagnosis (single/married/divorced or separated/widowed/unknown), smoking history at the time of diagnosis (yes/no/unknown), and patients’ birthplace (Cyprus districts) as well as the median and the interquartile range (IQR) of patients׳ age. Table 2, Table 3, Table 4, Table 5 present counts and percentages of newly diagnosed breast cancer cases by cancer grade (well differentiated/moderately differentiated/poorly differentiated/undifferentiated/ unknown), behaviour (in situ/invasive), stage (distant metastatic/in situ/locoregional invasive/unknown), and histological type at diagnosis (adenoid cystic carcinoma/carcinoma with apocrine features/carcinoma with medullary features/cribriform carcinoma/inflammatory carcinoma/invasive carcinoma of no special type/invasive lobular carcinoma/mesenchymal tumours/metaplastic carcinoma/mucinous carcinoma/Paget׳s disease/phyllodes tumour/rare variants/tubular carcinoma). Cases are also presented by ethnicity (Greek-Cypriot and Non-Greek-Cypriot). Armenians and Maronites belong to the Greek-Cypriot community but represent different ethnic/religious groups and given their small numbers were included along with the European Union (EU) foreigners and the non-EU foreigners in the non-Greek-Cypriot group. The ethnicity group “Unknown” is omitted. Subtypes of breast cancer classified in the categories of histological type used in the analysis are presented in Table 6. Table 7 gives breast cancer-related deaths by gender. Table 8, Table 9, Table 10, Table 11, Table 12, Table 13, Table 14, Table 15 show five-year net survival rates of breast cancer cases, based on cohort and period approaches, for 2004–2008 and 2009–2013, by age group, cancer stage, grade, and behaviour at diagnosis for all recorded cases (Table 8, Table 9, Table 10, Table 11) and for Greek-Cypriots only (Table 12, Table 13, Table 14, Table 15).

## 2 Experimental design, materials and methods

The data presented in this article were obtained from the Cyprus Cancer Registry and the Causes of Death Registry in the Health Monitoring Unit at the Ministry of Health of the Republic of Cyprus. The Cyprus Cancer Registry collects data from all public and private hospitals as well as from clinics within the Republic of Cyprus [1]. It was established in the context of the Middle East Cancer Consortium (MECC) Cancer Registry Project opened on 1st January 1998 aiming to standardize data items, definitions, and codes in order to ensure reliable comparisons. The reference date of diagnoses that are included in MECC for Cyprus is January 1, 1998. MECC is an intergovernmental organization established in 1996 in Geneva, Switzerland, by an agreement between Cyprus, Egypt, Israel, Jordan, and the Palestinian Authority. The aim of MECC is to raise cancer awareness and reduce the burden of cancer in Middle East [1].

The data in this article refer to the period between 2005 and 2013. The unprocessed data provided by the Health Monitoring Unit at the Ministry of Health were checked for duplicates. Duplicates were deleted from the dataset. Only patients residing in the controlled area by the Republic of Cyprus were included.

Overall, between 2005 and 2013, 4769 newly diagnosed breast cancer cases residing in the Republic of Cyprus were recorded in the Cyprus Cancer Registry. Their demographic characteristics (gender, age, ethnicity, marital status, smoking history, and birthplace) are presented in Table 1. The ethnicity of 69 cases was ‘Unknown’. The rest (4700) were classified into Greek-Cypriots and non-Greek Cypriots.

Counts and percentages of 4700 newly diagnosed breast cancer cases of known ethnicity are presented by cancer grade (Table 2), behaviour (Table 3), stage (Table 4), and histological type at diagnosis (Table 5). Table 6 shows breast cancer histological categories and their subtypes, which have been presented previously in Table 5, and the respective International Classification of Diseases for Oncology (ICD-O-3) morphological code adopted by the International Agency for Research on Cancer (IARC), World Health Organization (WHO) [2]. Table 7 shows breast cancer-related deaths by gender and the number of deaths attributable to breast cancer out of 1000 deaths among women and men, and in both sexes. Information on annual numbers of deaths in Cyprus were obtained from the report on mortality statistics between 2004 and 2014 [3].

Table 8, Table 9, Table 10, Table 11, Table 12, Table 13, Table 14, Table 15 present 5-year net survival rates for 2004–2008 and 2009–2013. Net survival rates are presented by age group (Table 8), cancer grade (Table 9), behaviour (Table 10), and stage at diagnosis (Table 11). The survival analysis included cases meeting the following criteria: (a) they were followed-up after their diagnosis and (b) their age was known at the time of diagnosis. The most recent entry with an updated cancer status was considered for patients recorded more than once in the registry. Five-year net survival rates were estimated using two different approaches: cohort-based and period [4], [5]. The cohort-based survival analysis involves cases diagnosed in 2004–2008 and their survival has been assessed in the 5 years (2009–2013) following the date of diagnosis. The period approach involves cases diagnosed over the period 2004–2013 but considers their survival experience in recent years i.e. over the period 2009–2013. Therefore, the period method provides more up-to-date estimates and quite closely predicts survival rates that will later be observed for cases diagnosed in 2009–2013. Net survival was estimated using the *stns* command in STATA version 14 [6]. Survival analyses were performed for all cases (Table 8, Table 9, Table 10, Table 11) and for cases of Greek-Cypriot ethnicity (Table 12, Table 13, Table 14, Table 15). Background mortality data used for net survival assessment were in the format of age-specific daily death rate for Cyprus and were retrieved by WHO [7]. All data were analyzed using STATA version 14 (StataCorp LP, College Station, Texas, USA).
