# Supplementary material for: Epidemiology of breast cancer in Cyprus: Data on newly diagnosed cases and survival rates
Source: Data Brief. 2018 May 19;19:353–69. doi: 10.1016/j.dib.2018.05.042 (PMC5993104; doi:10.1016/j.dib.2018.05.042)
Supplement: Supplementary file 1 — Supplementary material [file mmc1.docx]

**Conflict of Interest**

The authors confirm that there are no known conflicts of interest associated with this publication and there has been no significant financial support for this work that could have influenced its outcome.
